# Loss of HMGCS2 Enhances Lipogenesis and Attenuates the Protective Effect of the Ketogenic Diet in Liver Cancer

**DOI:** 10.3390/cancers12071797

**Published:** 2020-07-04

**Authors:** Yuan-Hsi Wang, Fat-Moon Suk, Yi-Jen Liao

**Affiliations:** 1 School of Medical Laboratory Science and Biotechnology, College of Medical Science and Technology, Taipei Medical University, Taipei 110, Taiwan; m609105001@tmu.edu.tw; 2 Division of Gastroenterology, Department of Internal Medicine, Wan Fang Hospital, Taipei Medical University, Taipei 116, Taiwan; 95351@w.tmu.edu.tw; 3 Department of Internal Medicine, School of Medicine, College of Medicine, Taipei Medical University, Taipei 110, Taiwan

**Keywords:** HMGCS2, ketogenic diet, lipogenesis, hepatocellular carcinoma

## Abstract

Hepatocellular carcinoma (HCC) is the most common primary malignant liver tumor with limited treatment. The ketogenic diet (KD) emerged as a metabolic therapy for cancer; however, the antitumor effect on HCC remains controversial. We previously reported that the ketogenesis rate-limiting enzyme, 3-hydroxymethylglutaryl-CoA synthase 2 (HMGCS2), was downregulated in most patients with HCC. The knockdown of HMGCS2 enhanced the proliferation and metastasis ability of HCC cells. However, the role of HMGCS2 in affecting KD-mediated metabolic effects remains unclear. Here, we report that KD feeding upregulates HMGCS2 expression and inhibits HCC tumor growth, while a reverse correlation between tumor size and HMGCS2 expression was observed. We found that HCC cells with HMGCS2 downregulation possess altered lipid metabolism that increases fatty acid, triglyceride, and cholesterol synthesis. Under KD feeding, a higher tumor growth rate was observed in HMGCS2 knockdown tumors, which had increased lipid synthesis-related marker expression and a positive correlation between lipid quantity and tumor weight. In conclusion, these results demonstrate that the downregulation of HMGCS2 attenuates the protective effect of the KD by shifting ketone production to enhance de novo lipogenesis in HCC. Our study elucidates a new molecular mechanism underlying the crosstalk between HMGCS2 expression and the KD in cancer treatment, which provides more information for precision medicine in developing personalized treatment strategies.

## 1. Introduction

Liver cancer incidence is rising faster than that for any other cancer [1]. In the 2018 global cancer statistic report, liver cancer was the sixth most commonly diagnosed cancer and the fourth leading cause of cancer death [2]. Hepatocellular carcinoma (HCC) is the most common primary malignant liver tumor, comprising 10–15% of cases [3]. Although much progress has been made in the clinical management of HCC in the past 10 years, therapeutic options and outcomes for patients with HCC are still limited [4]. Since 2017, different strategies for the treatment of patients with advanced HCC have been provided, including inhibitors of tyrosine kinase signaling, angiogenic molecules, and immune checkpoint inhibitors [4]. However, none of these therapies address the altered metabolic functions of HCC cells. Cancer cells exhibit atypical metabolism, which have higher glycolysis and lactate metabolism and defective mitochondrial ATP production, a phenomenon known as the Warburg effect [5]. A previous study revealed that glycolytic activity is upregulated by increasing hexokinase 2 activity and glucose transporter 1 (GLUT1) expression in HCC [6]. The Warburg effect facilitates the uptake and de novo synthesis of nutrients (including nucleotides, amino acids, and lipids) that are available for tumor growth [7]. Since the liver is essential to glucose, lipid, and cholesterol metabolism, pathological conditions, including HCC, also affect its metabolic function [8]. Therefore, targeting metabolic pathways may provide a novel therapeutic strategy for HCC.

The ketogenic diet (KD) is a high-fat, low-carbohydrate diet that was originally developed in the 1920s as a treatment for intractable epilepsy [9]. Recently, it has emerged as a potential metabolic therapy for cancer to reduce insulin secretion and switch to fat oxidation for fuel by converting fatty acids to ketone bodies [10]. Its use has been extensively studied in cell and animal models, and a growing number of clinical studies suggest that a KD could be a potent anticancer therapy [10,11,12,13]. For example, a previous study found that a KD inhibited gastric cancer growth and delayed the initiation of tumors in a nude mouse animal model [14]. In addition, it was reported that a KD protects healthy cells from damage by radiation or chemotherapy and enhances toxicity in cancer cells [15,16,17]. Clinical studies also demonstrate the potential benefits of a KD for patients with cancer [12,18]. In liver disease, a KD ameliorates nonalcoholic fatty liver disease [19,20]. In 2015, Poff et al. reported that mice fed with a KD have the lowest liver tumor burden after treatment with diethylnitrosamine (DEN) [21,22]. However, the same group in 2018 provided evidence for a minimal impact of a KD against liver cancer [23]. Altogether, the role of a KD in liver cancer remains unclear, and the factor that influences the antitumor effect of the KD on liver cancer needs to be addressed.

Ketones are organic compounds that are mainly produced in the liver [24]. When consuming a KD, cells are under low glucose and high-fat conditions. The increased fatty acid β-oxidation elevates acetyl-CoA production. Subsequently, excessive acetyl-CoA is used for ketone synthesis [25,26]. Notably, 3-hydroxymethyl glutaryl-CoA synthase 2 (HMGCS2) is the rate-limiting enzyme in the ketogenesis pathway [27]. Since ketogenesis is interwoven with metabolic pathways, including fatty acid oxidation, the citric acid cycle (TCA cycle), gluconeogenesis, de novo lipogenesis (DNL), and the biosynthesis of sterols [26], the role of HMGCS2 in affecting HCC metabolism is worthy of exploration. In 2014, Cotter et al. generated a ketogenic insufficient mouse model by the administration of antisense oligonucleotides targeted to HMGCS2. They found that the high-fat-diet feeding of HMGCS2-eliminated mice resulted in increased hepatic injury and inflammation and, therefore, suggested that ketogenesis may modulate nonalcoholic fatty liver disease [28]. Our group previously published that the loss of HMGCS2 enhanced the proliferation and metastasis of HCC [29]. However, the role of HMGCS2 in KD-mediated HCC metabolic treatment remains unknown. Therefore, this study aims to investigate the influence of HMGCS2 expression in response to KD treatment in HCC.

## 2. Results

### 2.1. Ketogenic Diet Feeding Inhibits HCC Tumor Growth by Increasing the Protein Expression of HMGCS2

**Figure 1:**
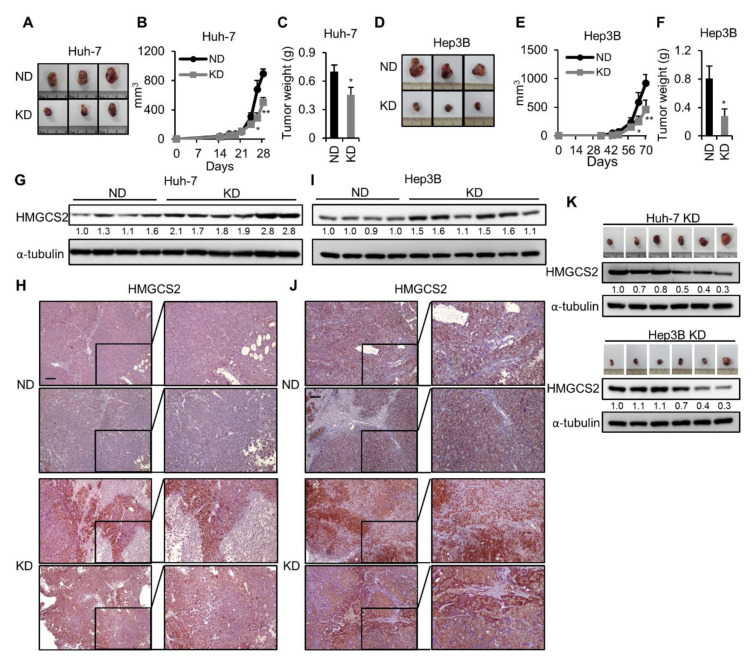
Mice fed a KD inhibited tumor growth, which was accompanied by increased HMGCS2 expression. Mice were fed an ND or KD two weeks before subcutaneous implantation of 5 × 10^6^ Huh-7 and Hep3B cells. *n* = 6/group. (**A**,**D**) Representative images of tumors from either feeding the normal diet (ND) or feeding the ketogenic diet (KD). (**B**,**E**) The tumor growth rate and (**C**,**F**) the tumor weight of each group. (**G**,**I**) Western blot analyses of HMGCS2 using protein lysates derived from tumors of the ND and KD groups. The whole blot images can be found in Figure S10. (**H,J**) Representative HMGCS2 IHC-stained sections of tumors from each treatment group. Scale bars: 0.1 mm. (**K**) Paired HMGCS2 western blotting images and tumor mass among the KD groups. The quantification of the western blot images was presented by using the ImageJ system. The Mann–Whitney U test was used to compare two independent groups. * *p* < 0.05; ** *p* < 0.01 vs. the black bar.

Here, we used a xenograft mouse model to analyze the KD effect on human HCC cell tumor formation. The results showed that the serum level of the ketone body was significantly elevated in the KD group without inducing any side effects, such as body weight and serum alanine aminotransferase (ALT) level changes (Figure S1A–C). In addition, we found that the tumor size (Figure 1A), tumor growth rate (Figure 1B), and tumor weight (Figure 1C) of the subcutaneously implanted Huh-7 cells were lower in KD fed mice. These phenomena were replicable in another HCC cell line, Hep3B (Figure 1D–F). HMGCS2 is the rate-limiting enzyme in ketogenesis pathway [27]; however, the change in HMGCS2 expression while feeding a KD is still unknown. Therefore, we compared the tumorous HMGCS2 expression between the normal diet (ND) and KD-fed mouse groups. The results showed that tumors from KD-fed mice expressed higher protein levels of HMGCS2 (Figure 1G,I). These results were also observed by using immunohistochemistry (IHC) staining (Figure 1H,J). In addition, the IHC staining demonstrated that HMGCS2 mainly localized in the cytoplasm. Next, we analyzed the correlation between the tumor size and HMGCS2 protein expression among the tumors of the KD-fed mice group. Surprisingly, we found a reverse correlation between tumor size and HMGCS2 protein expression in the KD-fed mouse group (Figure 1K). It was reported that inflammation was a hallmark of cancer that contributed to the development and progression of HCC [30]. In order to reveal the overall effect of the KD, we also evaluated the inflammatory changes in tumors of ND- and KD-fed mouse groups. The result showed that the mice fed with the KD did not affect the mRNA expression levels of interleukin-1 (IL-1), interleukin-1b (IL-1b), interleukin-8 (IL-8), and tumor necrosis factor-α (TNF-α) (Figure S1D). In addition, the histological analysis showed that the amounts of F4/80 were not significantly changed between ND-fed and KD-fed mouse groups (Figure S1E). Accordingly, these data suggested that the antitumor effect of KD was associated with the expression of HMGCS2.

### 2.2. Gene Expression Profiling of HMGCS2 Knockdown HCC Cells

**Figure 2:**
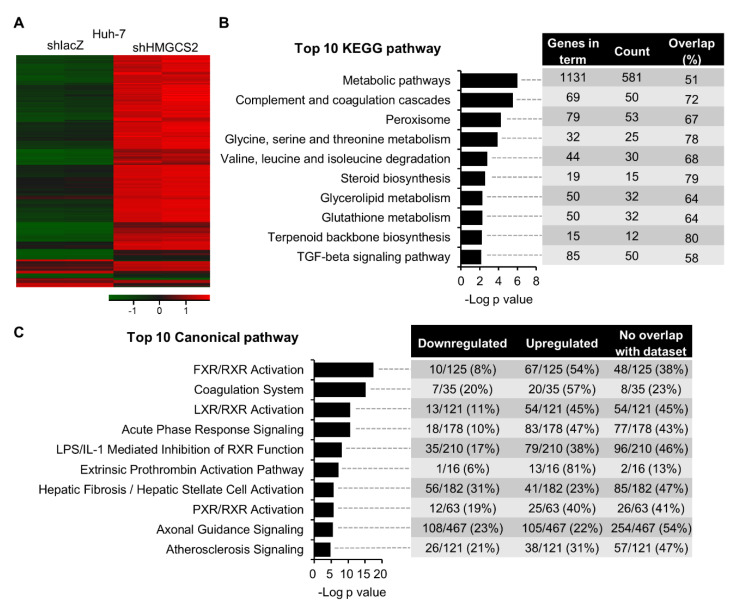
Detailed gene expression profile of HMGCS2 knockdown Huh-7 cells. (**A**) Heat map of the mRNA expression profile of shlacZ and shHMGCS2 cells based on mRNA microarray analysis. The differential expression of genes listed in the hierarchical clustering map was defined by the ratio of the expression in shHMGCS2 cells to that in shlacZ control cells as a log2 (fold change) of ≥ 2 (upregulation, red) or ≤ 0.5 (downregulation, green). (**B**) Top 10 Kyoto Encyclopedia of Genes and Genomes (KEGG) pathways. The height of the histogram corresponds to the relative expression value for a particular pathway that significantly changed. (**C**) Top 10 canonical pathways classified by IPA after HMGCS2 knockdown in Huh-7 cells.

To further understand how HMGCS2 regulates liver carcinogenesis, we compared global gene expression profiles between the HMGCS2 knockdown and shlacZ cells, which served as a negative control in the transfection experiment. The heat map data showed distinct gene expression patterns between Huh-7 shlacZ and shHMGCS2 cells (Figure 2A). In the KEGG pathway enrichment analysis, we found that the most affected pathways in HMGCS2 knockdown Huh-7 cells were metabolic pathways (KEGG pathway: hsa01100), including carbohydrate metabolism, amino acid metabolism, lipid metabolism, and etc. (Figure 2B). Ingenuity Pathway Analysis (IPA) showed that farnesoid X receptor/retinoid X receptor (FXR/RXR) activation and liver X receptor/retinoid X receptor (LXR/RXR) activation were mainly affected in the HMGCS2-downregulated Huh-7 cells (Figure 2C, Figures S2 and S3). These data implied that the loss of HMGCS2 function influences HCC cell metabolic pathways, especially lipid regulation.

### 2.3. Knockdown of HMGCS2 Enhanced Fatty Acid, Triglyceride, and Cholesterol Synthesis in HCC Cells

**Figure 3:**
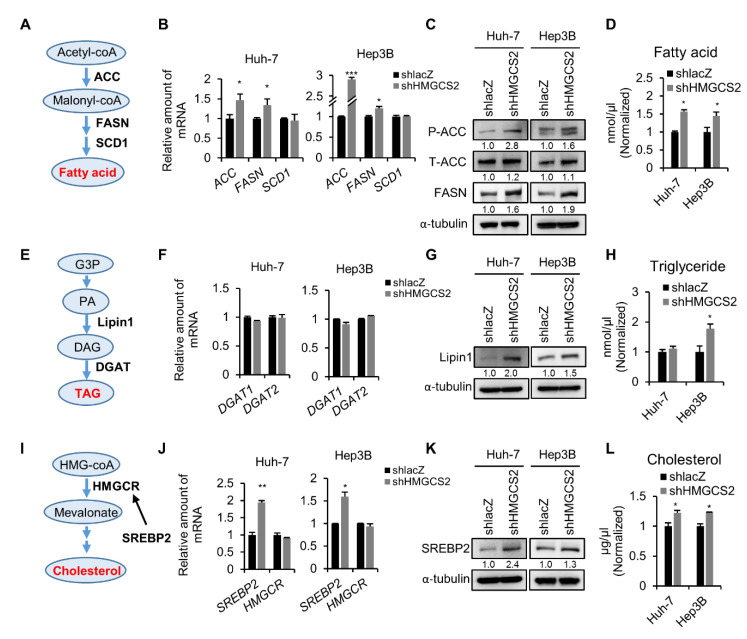
HMGCS2 downregulation affects fatty acid, triglyceride, and cholesterol synthesis in both Huh-7 and Hep3B cells. (**A**) Diagram of fatty acid synthesis. (**B**) qPCR analyses of ACC, FASN, and SCD1 gene expression. (**C**) Representative western blotting images of T/p-ACC, and FASN expression. (**D**) Intracellular fatty acids were measured using a colorimetric fatty acid quantification kit. (**E**) Diagram of triglyceride synthesis. (**F**) qPCR analyses of DGAT1 and DGAT2 gene expression. (**G**) Representative western blotting images of Lipin1 expression. (**H**) Intracellular triglyceride was confirmed by using a colorimetric triglyceride quantification kit. (**I**) Diagram of cholesterol synthesis. (**J**) qPCR analyses of SREBP2 and HMGCR gene expression. (**K**) Representative western blotting images of SREBP2 expression. (**L**) Intracellular cholesterol was measured by using a colorimetric cholesterol quantification kit. The quantification of the western blot images was presented by using the ImageJ system and the whole blot images can be found in Figure S11. The Mann–Whitney U test was used to compare two independent groups. * *p* < 0.05; ** *p* < 0.01; *** *p* < 0.001 vs. the black bar. G3P: glycerol-3-phosphate; PA: phosphatidic acid, DAG: diacylglycerol, TAG: triacylglycerol.

Hepatic fatty acid, triglyceride, and cholesterol metabolism are important factors for maintaining the homeostasis of the lipid pool [31]. Dysregulation of lipid metabolism may trigger cancer development, including HCC [32,33]. Thus, we further clarified the regulatory mechanisms of HMGCS2 expression on the fatty acid synthesis, triglyceride synthesis, and cholesterol synthesis. For fatty acid biosynthesis (Figure 3A), acetyl-CoA is converted into malonyl-CoA by acetyl-CoA carboxylase (ACC). The condensation of acetyl-CoA and malonyl-CoA by fatty acid synthase (FASN) leads to the production of palmitic acid. A double bond is introduced by stearoyl-CoA desaturase (SCD1) to generate monounsaturated fatty acids. We demonstrated that the knockdown of HMGCS2 significantly increased the mRNA expression levels of ACC and FASN (Figure 3B) and the protein expression levels of phosphorylated ACC and total FASN (Figure 3C). The results of intracellular fatty acid quantification showed that the knockdown of HMGCS2 in both Huh-7 and Hep3B cells significantly increased the fatty acid amount (Figure 3D). For triglyceride biosynthesis (Figure 3E), glycerol-3-phosphate undergoes esterification to form phosphatidic acid. Phosphatidic acid phosphohydrolase (PAPase, which is also known as Lipin), hydrolyzes phosphate to form diacylglycerol. Diacylglycerol acyltransferase (DGAT) catalyzes the third esterification to form triacylglycerol [34]. Although HMGCS2 knockdown cells did not influence the mRNA expression levels of DGAT1 and DGAT2 (Figure 3F), the protein expression level of Lipin1 was increased in both Huh-7 and Hep3B shHMGCS2 cells (Figure 3G). Triglyceride quantification assays showed that Hep3B shHMGCS2 cells possessed higher triglyceride levels than the control (Figure 3H). In cholesterol biosynthesis (Figure 3I), sterol regulatory element-binding protein 2 (SREBP2) is the primary transcription factor that induces the expression of 3-hydroxy3-methylglutaryl-CoA reductase (HMGCR). HMGCR is the key enzyme that converts 3-methylglutaryl-3-hydroxy-CoA (HMG-CoA) to mevalonate, and subsequent reactions result in the production of cholesterol. The mRNA and protein expression levels of SREBP2 were elevated in the HMGCS2 knockdown cells, while the HMGCR mRNA expression remained unchanged (Figure 3J,K). The cholesterol quantification results showed that the knockdown of HMGCS2 enhanced the cholesterol amount in both Huh-7 and Hep3B cells (Figure 3L). These data indicate that the loss of HMGCS2 results in the dysregulation of lipid synthesis in HCC cells. On the other hand, HMGCS2 overexpression did not affect fatty acid, triglyceride, and cholesterol synthesis in either Huh-7 or Hep3B cells (Figure S4).

A previous study revealed that glycolytic activity is upregulated in HCC, a process that refers to the Warburg effect, and thereby facilitates uptake and de novo synthesis of lipids [7]. Therefore, the glycolysis pathway was also evaluated. The results showed that the mRNA expression of glycolysis pathway-related markers (phosphofructokinase (PFK), pyruvate kinase (PK), and lactate dehydrogenase (LDH)) were not significantly changed in the HMGCS2-downregulated and HMGCS2-upregulated cells compared with the relative control (Figure S5A). In addition, the protein expression level of glyceraldehyde 3-phosphate dehydrogenase (GAPDH) was not significantly changed in different HMGCS2 expression cells (Figure S5B). These results suggest that knockdown of HMGCS2 upregulates de novo lipogenesis, thereby leading to the accumulation of lipids in HCC cells without affecting glycolysis signaling. We thus hypothesized that HMGCS2 expression might be a key factor that influences the antitumor effect of KD therapy.

### 2.4. HMGCS2 Knockdown HCC Cells Possessed Increased Tumor Growth Ability under Ketogenic Diet Feeding

**Figure 4:**
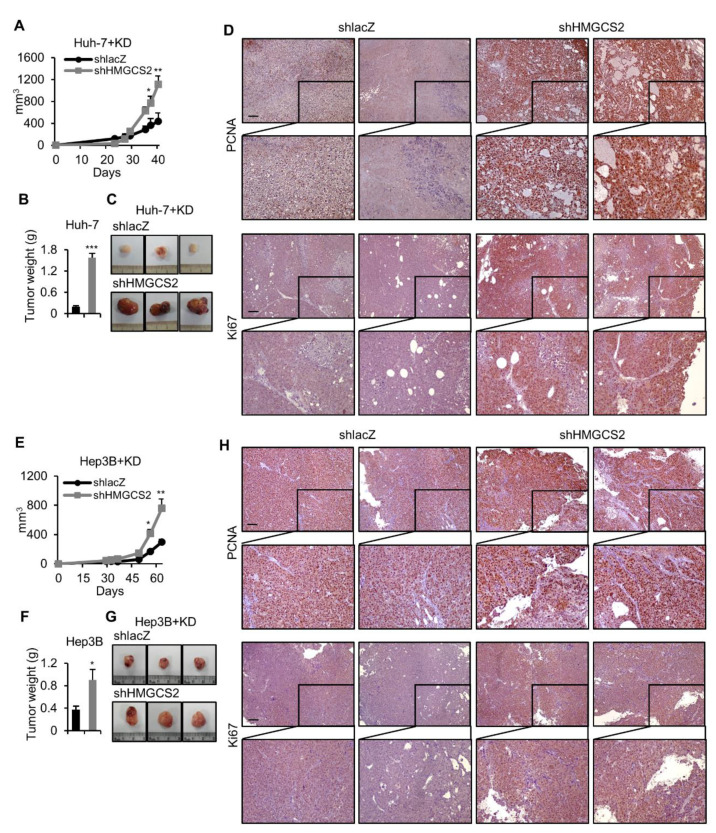
The knockdown of HMGCS2 enhanced tumor growth under KD feeding. (**A**,**E**) The tumor growth rate and (**B**,**F**) the tumor weight from mice fed either an ND or KD. Huh-7+KD: *n* = 7/group. Hep3B+KD: *n* = 5/group. (**C**,**G**) Representative images of tumors from each group. (**D**,**H**) Representative PCNA and Ki67 IHC staining of tumors from each treatment group. The Mann–Whitney U test was used to compare two independent groups. * *p* < 0.05; ** *p* < 0.01; *** *p* < 0.001 vs. the black bar.

To further study the genetic effect of HMGCS2 on the antitumor ability of a KD in vivo, HMGCS2 knockdown Huh-7, and Hep3B cells were subcutaneously injected into NOD/SCID mice and maintained on a KD from the first day of cell implantation. We observed that HMGCS2 knockdown cells possessed significantly greater tumor growth (Figure 4A,E) and enhanced tumor weight and tumor size (Figure 4B,C,F,G) compared with the control group. The histological analysis identified that under KD feeding, HMGCS2 knockdown tumors expressed a higher nuclear signal level of proliferation markers, both PCNA and Ki-67, compared with that of the shlacZ control tumors (Figure 4D,H). We further investigated whether the tumor inflammation was influenced by HMGCS2 expression in response to KD treatment. The result showed that the mRNA expression levels of IL-1, IL-1b, IL-8, and TNF-α were not significantly changed between Huh-7 HMGCS2 knockdown and shlacZ control tumors. In Hep3B knockdown tumors, IL-1b, IL-8, and TNF-α were not changed, while IL-1 was mildly increased compared with that of the shlacZ control tumors (Figure S6A). In addition, the histological analysis showed that the amounts of F4/80 were not significantly changed between HMGCS2 knockdown and shlacZ control tumors in both Huh-7 and Hep3B tumors (Figure S6B). We also evaluated the tumor growth ability of HMGCS2 overexpression under KD feeding. The results showed that the tumor weight in the HMGCS2 overexpression group was slightly lower than that in the control group (Figure S6C,D), while the amounts of PCNA and Ki-67 nuclear signal were not significantly changed between the HMGCS2 overexpression and the eGFP control tumors (Figure S6E). In addition, the mRNA expression levels of IL-1, IL-1b, IL-8, and TNF-α; the histological analysis of F4/80 expression were not significantly changed between HMGCS2 overexpression and eGFP control tumors (Figure S6F,G). Collectively, HMGCS2 downregulated tumors are resistant to the antitumor effect of the KD.

### 2.5. Tumors from HMGCS2 Knockdown HCC Cells Showed Increased Lipid Synthesis-related mRNA and Protein Markers under the Ketogenic Diet

**Figure 5:**
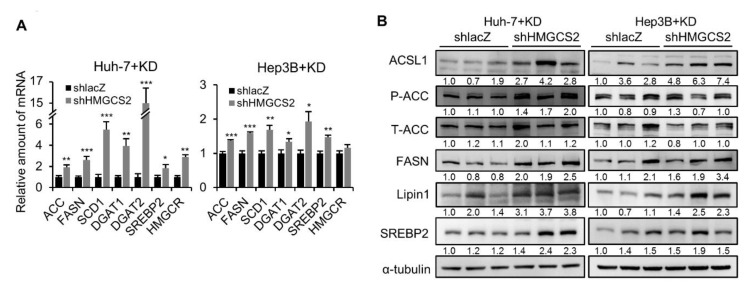
HMGCS2-downregulated tumors had increased expression of lipogenesis-related markers under KD feeding. (**A**) qPCR analyses of ACC, FASN, SCD1, DGAT1, DGAT2, SREBP2, and HMGCR gene expression and (**B**) western blot analyses of ACSL1, T/P-ACC, FASN, Lipin1, and SREBP2 expression of KD fed shlacZ and shHMGCS2 tumors. The quantification of the western blot images was presented by using the ImageJ system, and the whole blot images can be found in Figure S12. The Mann–Whitney U test was used to compare two independent groups. * *p* < 0.05; ** *p* < 0.01; *** *p* < 0.001 vs. the black bar.

Next, we further investigated whether HMGCS2 knockdown tumors are critical in affecting lipogenesis under KD feeding. Impressively, loss of tumorous HMGCS2 expression significantly increased the mRNA levels of key genes involved in fatty acid synthesis (ACC, FASN, and SCD1), triglyceride synthesis (DGAT1 and DGAT2), and cholesterol synthesis (SREBP2 and HMGCR) (Figure 5A). Similarly, lipid synthesis-related proteins, such as ACSL1, FASN, Lipin1, and SREBP2, were increased in both Huh-7 and Hep3B shHMGCS2 tumors (Figure 5B). On the other hand, we also evaluated lipid synthesis markers in HMGCS2-overexpressing tumors. The results showed that the mRNA and protein expression levels of key components of fatty acid synthesis, triglyceride synthesis, and cholesterol synthesis were not significantly changed between the HMGCS2 overexpression and eGFP control groups (Figure S7). These data indicated that the antitumor effect of KD was reversed in HMGCS2-downregulated HCC tumors due to the increase in de novo lipogenesis that promoted tumor growth.

### 2.6. Tumors from HMGCS2 Knockdown Cells Showed Increased Lipids Amount (Fatty Acids, Triglycerides, and Cholesterol) and That Was Correlated with the Tumor Weight

**Figure 6:**
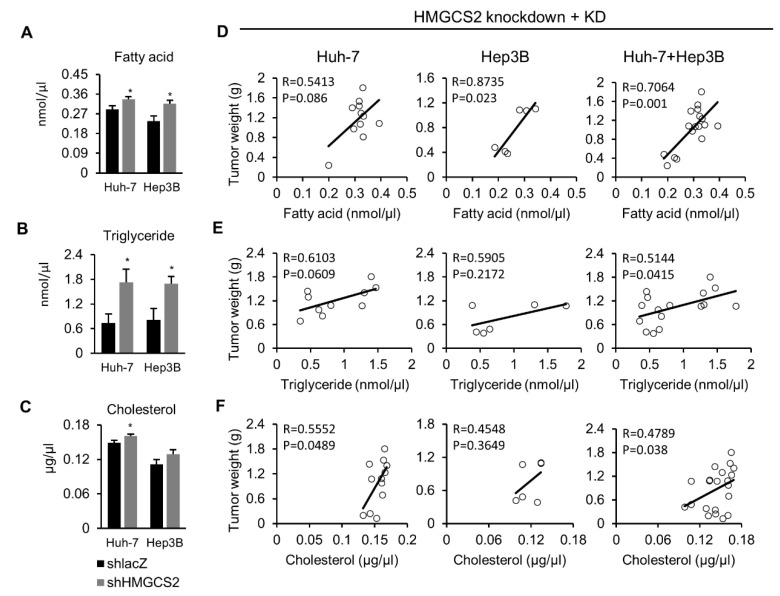
HMGCS2-downregulated tumors showed elevated lipid content and positively correlated with tumor weight. (**A**–**C**) The fatty acid, triglyceride, and cholesterol in the tumor mass. (**D**–**F**) Pearson correlation between each lipid content and tumor weight in the indicated groups. The Mann–Whitney U test was used to compare two independent groups. * *p* < 0.05 vs. the black bar.

To further confirm our findings (Figure 5), we used commercial kits to quantify the amounts of fatty acids, triglycerides, and cholesterol in the tumor mass. The results showed that HMGCS2-downregulated HCC tumors expressed higher levels of fatty acids, triglycerides, and cholesterol than shlacZ control tumors under KD feeding (Figure 6A–C). Among the three different lipid species (fatty acids, triglycerides, and cholesterol), the increase in triglycerides was most significant compared with that of the shlacZ control (Figure 6B). Moreover, we further analyzed the correlations between each lipid species and tumor weight among HMGCS2 knockdown tumors. As shown in Figure 6D, a positive correlation was detected between tumorous fatty acids and tumor weight in Huh-7 (Pearson correlation: R = 0.5413; *p* = 0.086), Hep3B (Pearson correlation: R = 0.8735; *p* = 0.023), and both cell lines (Pearson correlation: R = 0.7064; *p* = 0.001). In Figure 6E, we found a positive correlation between tumorous triglyceride and tumor weight in Huh-7 (Pearson correlation: R = 0.6103; *p* = 0.0609), Hep3B (Pearson correlation: R = 0.5905; *p* = 0.2172), and both cell lines (Pearson correlation: R = 0.5144; *p* = 0.0415). In Figure 6F, a positive correlation was also observed between tumorous cholesterol and tumor weight in Huh-7 (Pearson correlation: R = 0.5552; *p* = 0.0489), Hep3B (Pearson correlation: R = 0.4548; *p* = 0.3649), and both cell lines (Pearson correlation: R = 0.4789; *p* = 0.038). On the other hand, the amounts of fatty acids, triglycerides, and cholesterol in the tumor mass were not significantly different between the HMGCS2 overexpression group and the eGFP control group (Figure S8A). In addition, there was no correlation between the three different lipid species (fatty acids, triglycerides, and cholesterol) and tumor weight (Figure S8B).

## 3. Discussion

In recent years, the KD has gained growing attention as an approach to target metabolic differences between normal and cancer cells [13]. A KD restricts glucose availability and limits the Warburg-type metabolism in cancer cells [10]. Generally, most clinical and preclinical studies have demonstrated a beneficial anticancer effect of a KD [10,35]. However, some studies of gliomas and medulloblastoma showed no impact of a KD on reducing tumor volume [36,37]. Moreover, the protumor effect of a KD was found in renal cancer [38]. Clearly, there are tissue-specific or genetic components that might determine whether tumors are sensitive to KD therapy. Therefore, this study investigates the interplay between a KD under different HMGCS2 expression conditions in the HCC tumors.

In this study, we showed that the tumor size, tumor growth rate, and tumor weight were lower under KD feeding (Figure 1A–F). In addition, the expression of HMGCS2 was markedly upregulated in mice fed a high-fat KD (Figure 1G–J). Another study showed that high-fat-diet-induced ketogenesis occurs through upregulating HMGCS2 expression [39]. Therefore, it is obvious that the increasing demand for cells to generate ketone bodies leads to the upregulation of the ketogenesis rate-limiting enzyme HMGCS2 expression. By consuming a high-fat diet, including the KD, allows the cell to upregulate HMGCS2 expression and convert fatty acids to ketone bodies. Previously, we reported that HMGCS2 protein expression is significantly reduced in liver tissues from HCC patients. Moreover, the expression of HMGCS2 was negatively correlated with the pathological grade and clinical stage [29]. We found that HMGCS2 controls the proliferation and migration abilities of liver cancer cells by regulating the apoptosis, c-Myc/cyclin D1, and EMT signaling pathways and acts in a ketone-dependent manner [29]. Thus, using a KD will increase HMGCS2 expression, and thereby, enhance ketone production to inhibit tumor growth. However, in other liver cancer-related studies, DEN-treated C57BL/6J mice fed a KD yielded diverse conclusions. Two studies showed that a KD had an antitumor effect [21,22], while one study showed a minimal effect on decreasing DEN-induced liver tumor growth [23]. We proposed that it is the different HMGCS2 expression levels that lead to the different outcomes of the treatment. Indeed, we found that the tumor sizes in KD-fed mice were negatively correlated with the HMGCS2 protein expression level (Figure 1K). In this study, our data implied that HMGCS2 expression is an important factor that influences the therapeutic effect of a KD in HCC.

To understand how HMGCS2 regulates liver carcinogenesis, we performed global gene expression analysis (Figure 2). We found that the knockdown of HMGCS2 in HCC cells significantly influenced metabolic pathway regulation, especially in lipid synthesis (Figure 2 and Figure 3). Indeed, HMGCS2 has been proposed as a moonlighting protein that acts as a coactivator to interact with peroxisome proliferator-activated receptor-α (PPARα) and upregulates the transcription of fatty acid oxidation by nuclear translocation [40,41]. A previous study demonstrated that HMGCS2 expression was required for fatty acid oxidation in a hepatoma cell line [42]. In contrast, Cotter et al. [28] revealed that PPARα-targeted fatty acid oxidation was induced in the absence of hepatic HMGCS2. These data imply that HMGCS2 plays an important role in lipid regulation. To gain more insight into the gene-diet interaction in the treatment of HCC, we showed that HMGCS2 knockdown HCC tumors had increased tumor size when a KD was used (Figure 4). In addition, tumors with HMGCS2 knockdown possessed increased lipid synthesis-related mRNA and protein markers under KD feeding (Figure 5). The increased lipid quantity of fatty acids, triglycerides, and cholesterol in the tumor mass was correlated with the tumor weight (Figure 6). Taken together, the loss of HMGCS2-mediated ketogenesis may lead to the poor outcome of HCC progression while using a KD as a therapeutic approach. A previous study found that ketogenesis may modulate fatty liver disease [28]. Mice lacking HMGCS2 expression caused insufficient ketogenesis under high-fat diet feeding, which promoted the development of nonalcoholic fatty liver diseases [28,43]. In many cancers, aberrant fatty acid metabolism has been observed, and de novo lipogenesis is often upregulated in solid tumors [33,44]. A recent study found that elevated liver de novo lipogenesis plays a key role in the development of HCC [32,45]. Previously, we reported that HMGCS2 downregulation enhanced the proliferation and migration abilities of liver cancers [29]. With our new findings, it is possible that not only decreased ketone production but also upregulated de novo lipogenesis in HMGCS2 knockdown HCC cells enhanced tumor growth.

**Figure 7:**
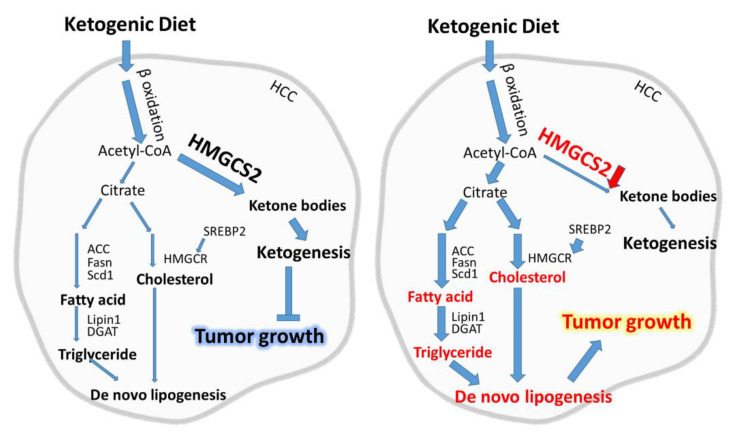
HMGCS2 downregulation increased de novo lipogenesis, which reversed the antitumor effect of a KD. In HCC tumors with HMGCS2 expression (left panel), KD supplementation increases ketogenesis mediated by HMGCS2, and therefore, inhibits HCC tumor growth. In the absence of HMGCS2 (right panel), the HCC tumors will be under the condition of ketogenic insufficiency. Under this circumstance, the use of a KD will increase de novo lipogenesis and ultimately trigger HCC tumor growth.

Our data could be concluded with a model (Figure 7) in which the change in lipid metabolism occurred while HMGCS2 was downregulated. Under normal conditions (Figure 7 left), feeding with a KD increases fatty acid β-oxidation and the production of acetyl-CoA, which is further used for ketone synthesis [10]. Therefore, we hypothesized that the expression of the ketogenesis rate-limiting enzyme HMGCS2 may be upregulated, and thereby, increase ketone production to inhibit tumor growth. Our data reveal that mice fed a KD inhibited tumor growth, which was accompanied by increased HMGCS2 expression (Figure 1). However, our previous study showed that HMGCS2 was downregulated in some HCC tumors [29]. Therefore, we speculated that the expression of HMGCS2 might lead to the different outcomes of HCC progression while using a KD as a therapeutic approach. Our data demonstrated that when there was a loss of HMGCS2, the convergence of ketogenic insufficiency with exposure to a high-fat composed KD resulted in additional metabolic sequelae (Figure 7 right). Our results indicate that the downregulation of HMGCS2 in HCC tumors reversed the antitumor effect of a KD by increasing de novo lipogenesis. Our study elucidates a new molecular mechanism underlying the crosstalk between HMGCS2 expression and a KD in cancer treatment, which provides more information for precision medicine in developing personalized anti-liver cancer strategies.

## 4. Materials and Methods

### 4.1. Animal Study

All animal procedures were approved by the Institutional Animal Care and Use Committee of Taipei Medical University (LAC-2019-0186, May 2019 approved). Seven- to eight-week-old male NOD/SCID mice were purchased from the Taiwan National Laboratory Animal Center and used for xenograft study. For in vivo tumorigenesis assays, 5 × 10^6^ Hep3B (parental, shlacZ and shHMGCS2) and Huh-7 (parental, eGFP, and HMGCS2) cells were subcutaneously injected into NOD/SCID mice (5–7 mice for each group). All mice were maintained on a standard chow diet (no. 5001, LabDiet, St. Louis, MO, USA) or a ketogenic diet (5TJQ, TestDiet, Richmond, IN, USA). Tumor size was measured three times a week with a Vernier caliper, and tumor volume was determined with the formula TV = (L × W^2^)/2, where L is the length and W is the width of the tumors. Tumor weight was measured using an electronic balance. This study is compliant with all relevant ethical regulations regarding animal research. The samples used in protein and RNA analyses were frozen in liquid nitrogen and stored at −80 ℃, while those used for IHC staining were fixed in 10% formalin.

### 4.2. Immunohistochemistry (IHC) Staining and Biochemical Blood Parameters

Tumors were fixed in freshly prepared 10% formalin. Paraffin-embedded tumor sections were incubated with antibodies against PCNA, Ki-67 (1:150; GenScript, Piscataway, NJ, USA), and HMGCS2 (1:200, Abcam, Cambridge, MA, USA) and detected using the Universal LSABTM2 kit (DakoCytomation, Carpinteria, CA, USA) according to the manufacturer’s instructions. All sections were investigated by a light microscope (Olympus CKX41, Olympus Corp., Tokyo, Japan). Serum alanine aminotransferase (ALT) was measured with a biochemical analyzer (VetTest™, IDEXX, Westbrook, ME, USA).

### 4.3. Cell Culture and Viral Infection

293T (purchased from ATCC, Manassas, VA, USA), Hep3B (purchased from BCRC, Hsinchu, Taiwan, No. 60434) and Huh-7 cells were cultured in DMEM supplemented with 10% FBS, 2 mM glutamine, 0.1 mM nonessential amino acids, and 100 U/mL penicillin and streptomycin. The establishment of cell lines with stable HMGCS2 overexpression and knockdown was previously described [29]. In brief, Hep3B and Huh-7 cells were infected with transfected-293T-cell-generated lentivirus in a medium containing polybrene (8 μg/mL). After 24 h of infection, 1 μg/mL puromycin was added to select the stable cells. Stable cells (Hep3B/Huh-7 shlacZ, shHMGCS2, eGFP, and HMGCS2) were cultured in Dulbecco’s modified Eagle’s medium (DMEM) supplemented with 10% fetal bovine serum (FBS) and 1 μg/mL puromycin (Sigma Aldrich, St Louis, MO, USA). The origin of LacZ was from E. coli β-galactosidase, and shlacZ did not target any human or mouse genes. Therefore, it was used as a negative control. HMGCS2 protein expression in cell lines with stable HMGCS2 overexpression and knockdown were verified by western blotting (Figure S9).

### 4.4. Gene Expression Profiling

The mRNA profiles were analyzed using Human OneArray Plus (Phalanx Biotech, Hsinchu, Taiwan). Total RNA was extracted from Huh-7 shlacZ and Huh-7 shHMGCS2 cells. Whole-genome gene expression was measured in these samples using OneArray Plus chips (Phalanx Biotech Group). Hierarchical clustering was performed using Cluster 3.0 (http://bonsai.hgc.jp/~mdehoon/software/cluster/). The differential expression of genes listed in the hierarchical clustering map was defined by the ratio of the expression in shHMGCS2 cells to that in shlacZ control cells as a log2 (fold change) of ≥2 (upregulation) or ≤0.5 (downregulation). The gene expression patterns in different pathways were analyzed using the KEGG pathway database (https://www.genome.jp/kegg/pathway.html).

### 4.5. Colorimetric Free Fatty Acid Assay

Free fatty acids were analyzed by using a commercial Colorimetric Free Fatty Acid Quantification Assay Kit (BioVision, Mountain View, CA, USA). In brief, the free fatty acids from 10^6^ cells or 10-mg tissue samples were extracted by homogenization with chloroform-TritonX-100. Acyl-CoA synthesis reagent was added to convert fatty acids to their CoA derivatives, which were subsequently oxidized by the commercial enzyme and substrate mix with the concomitant generation of color. The absorbance at 570 nm was measured.

### 4.6. Colorimetric Triglyceride Assay

Triglycerides were analyzed using a commercial Colorimetric Triglyceride Quantification Assay Kit (BioVision, Mountain View, CA, USA). Briefly, 10^7^ cells or 100-mg tissue samples were homogenized in 1 mL of a solution containing 5% NP-40 in water. The triglyceride was converted to free fatty acid and glycerol by adding lipase. The glycerol was then oxidized by the commercial enzyme and substrate mix to generate a product that reacts with the probe to generate color (spectrophotometry at OD 570 nm).

### 4.7. Colorimetric Total Cholesterol Assay

Total cholesterol production was analyzed by using a commercial Colorimetric Total Cholesterol Quantification Assay Kit (BioVision, Mountain View, CA, USA). In brief, cholesterol from 10^6^ cells or 10-mg tissue samples was extracted with chloroform:Isopropanol:NP-40 (7:11:0.1) in a homogenizer. The extracted cholesterol was then oxidized by cholesterol oxidase to yield H_2_O_2_, which reacted with a commercial probe to produce color (OD 570 nm).

### 4.8. Colorimetric β-Hydroxybutyrate Assay

Ketone body production was analyzed by using a commercial colorimetric β-hydroxybutyrate quantification assay kit (BioVision, Mountain View, CA, USA). Briefly, serum collected from the ND and KD groups was spun-filtered using a 10 Kd spin column (BioVision, Mountain View, CA, USA). Fifty microliters of filtered serum was added to the commercial enzyme and substrate mix in a 96-well plate. After incubation at room temperature for 30 min with protection from light, the absorbance at 450 nm was measured.

### 4.9. Western Blotting (WB)

WB was performed as previously described [29]. Each experiment was successfully carried out three times, as indicated. The following antibodies were used for WB: anti-HMGCS2 antibody (1:1000, Abcam, Cambridge, MA, USA), anti-ACC antibody (1:1000, Cell Signaling, Beverly, MA, USA), anti-phospho (S79) ACC antibody (1:1000, Cell Signaling, Beverly, MA, USA), anti-FASN antibody (1:1000, Cell Signaling, Beverly, MA, USA), anti-Lipin1 antibody (1:1000, Cell Signaling, Beverly, MA, USA), anti-SREBP2 antibody (1:1000, Cell Signaling, Beverly, MA, USA), anti-ACSL1 antibody (1:1000, Cell Signaling, Beverly, MA, USA), anti-GAPDH antibody (1:2000, Proteintech, Chicago, IL, USA) anti-α-tubulin antibody (1:5000, Sigma-Aldrich, St. Louis, MO, USA). The ImageJ software (National Institutes of Health, Bethesda, MD, USA) was used to quantify the images.

### 4.10. RNA Extraction and Quantitative RT-PCR

Total RNA was extracted via TRIzol Reagent (Ambion, Carlsbad, CA, USA). Total RNA (2 μg) was subjected to reverse transcription with High-Capacity cDNA Reverse Transcription Kits (Applied Biosystems, Carlsbad, CA, USA), as previously described [29]. To determine the relative mRNA level, qPCR was performed using KAPA SYBR FAST qPCR Master Mix (KAPA Biosystems, Boston, MA, USA) and gene expression was normalized to that of GAPDH. The primers used for qPCR are listed as follows: HMGCS2, forward, 5′-AAGTCTCTGGCTCGCCTGATGT-3′, reverse, 5′-TCCAGGTCCTTGTTGGTGTAGG-3′. ACC, forward, 5′-CCAGGCCATGTTGAGACGCT-3′, reverse, 5′-ATCACAGAGCGGACGCCATC-3′. FASN, forward, 5′-CTTCCGAGATTCCATCCTACGC-3′, reverse, 5′-TGGCAGTCAGGCTCACAAACG-3′. SCD1, forward, 5′-AGTTCTACACCTGGCTTTGG-3′, reverse, 5′-GTTGGCAATGATCAGAAAGAGC-3′. DGAT1, forward, 5′-GCGGCTGTGGTCTTACTG-3′, reverse, 5′-CTGCGGCACCATGAGTT-3′. DGAT2, forward, 5′-GGTCCTGTCCTTCCTTGT-3′, reverse, 5′-AGTTGCCTGCCAGTGTAG-3′. SREBP2, forward, 5′-CCCTTCAGTGCAACGGTCATTCAC-3′, reverse, 5′-TGCCATTGGCCGTTTGTGTC-3′. HMGCR, forward, 5′-GATGGGAGGCCACAAAGAG-3′, reverse, 5′-TTCGGTGGCCTCTAGTGAGA-3′.GAPDH, forward, 5′-TCACCACCATGGAGAAGGC-3′, reverse, 5′-GCTAAGCAGTTGGTGGTGCA-3′.

### 4.11. Statistical Analysis

The in vitro experiment results are expressed as the means ± standard deviations (SDs), while the in vivo experiment results are expressed as the means ± standard error of the mean (SEM). The data were analyzed by nonparametric tests in the SPSS v20.0 software (SPSS Inc., Chicago, IL, USA). The Mann–Whitney U test was used to compare two independent groups. Pearson correlation coefficient was used to explore relationships between two variables. Differences were considered statistically significant at *p* < 0.05.

## 5. Conclusions

Our study used cellular experiments and animal models to demonstrate that the downregulation of HMGCS2 attenuates the protective effect of the ketogenic diet, which is a shift from ketogenesis to enhancing the de novo lipogenesis pathways in HCC. Taken together, our data suggest that HMGCS2 is an important factor that needs to be considered before using a ketogenic diet as a therapeutic approach for patients with HCC.

## Supplementary Materials

The following are available online at https://www.mdpi.com/2072-6694/12/7/1797/s1, Figure S1: KD feeding did not induce any side effects in mice, Figure S2: KEGG enrichment pathway: FXR/RXR activation, Figure S3: KEGG enrichment pathway: LXR/RXR activation, Figure S4: HMGCS2 overexpression did not affect fatty acid, triglyceride, and cholesterol synthesis in both Huh-7 and Hep3B cells, Figure S5: HMGCS2 expression did not affect the glycolysis signaling pathway in HCC cells, Figure S6: HMGCS2 overexpression did not affect tumor growth in KD-fed mice and HMGCS2 expression did not influence the tumor inflammation, Figure S7: HMGCS2-overexpressed tumors did not influence expression of lipogenesis-related markers under KD feeding, Figure S8: HMGCS2-overexpressed tumors did not alter lipid content and showed no correlation with tumor weight, Figure S9: Establishment of cell lines with stable HMGCS2 overexpression and knockdown, Figure S10: Original unedited pictures and protein quantification of Figure 1, Figure S11: Original unedited pictures and protein quantification of Figure 3, Figure S12: Original unedited pictures and protein quantification of Figure 5, Figure S13: Original unedited pictures and protein quantification of Figure S4, Figure S14: Original unedited pictures and protein quantification of Figure S5, Figure S15: Original unedited pictures and protein quantification of Figure S7, Figure S16: Original unedited pictures and protein quantification of Figure S9.
