# Supplementary material for: Loss of HMGCS2 Enhances Lipogenesis and Attenuates the Protective Effect of the Ketogenic Diet in Liver Cancer
Source: Cancers (Basel). 2020 Jul 4;12(7):1797. doi: 10.3390/cancers12071797 (PMC7408319; doi:10.3390/cancers12071797)
Supplement: Supplementary file 1 [file cancers-12-01797-s001.pdf]

Supplementary Materials: Loss of HMGCS2 Enhances Lipogenesis and Attenuates the Protective Effect of the Ketogenic Diet in Liver Cancer

Yuan-Hsi Wang, Fat-Moon Suk and Yi-Jen Liao

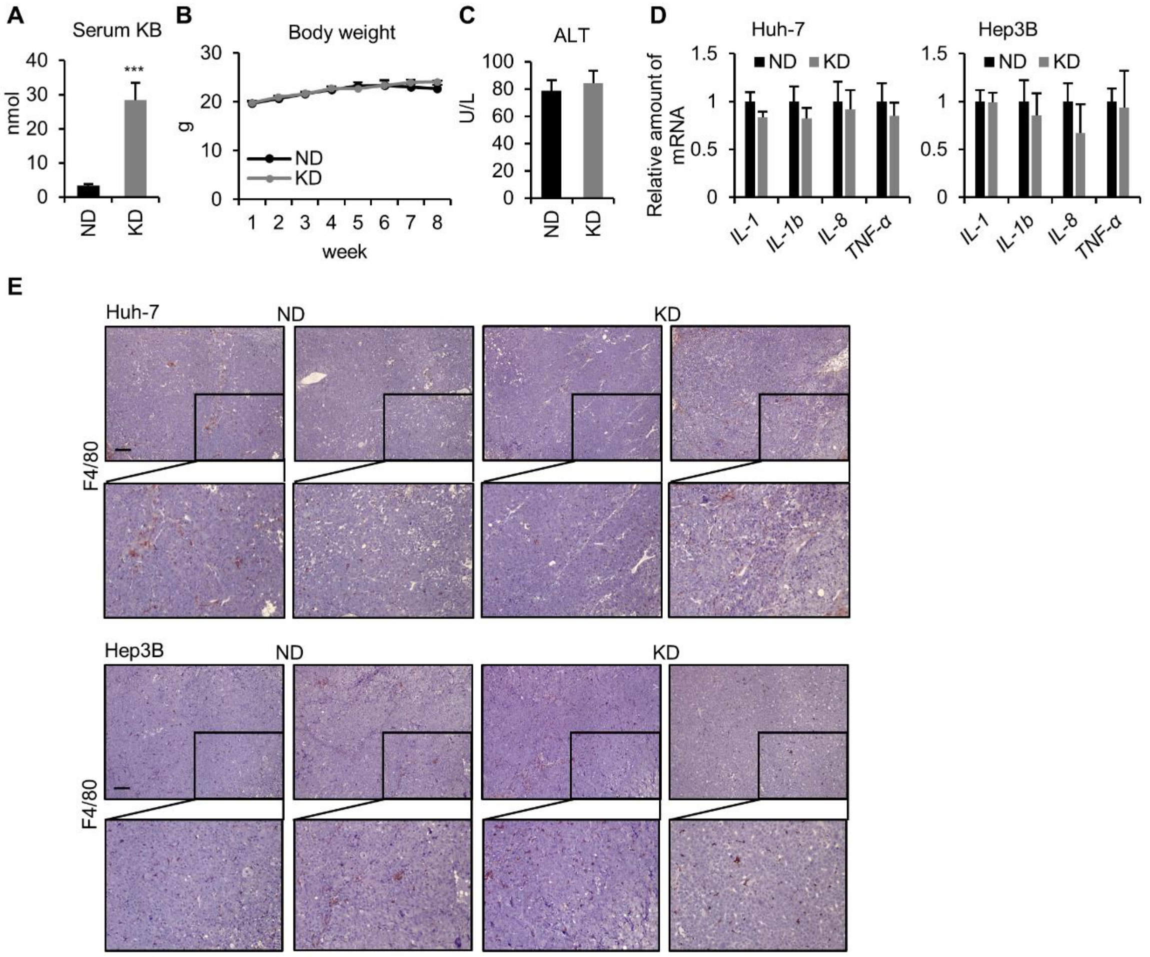

Figure S1. KD feeding did not induce any side effects in mice. (**A**) Serum ketone body (KB) concentration, (**B**) mice body weight, and (**C**) serum ALT levels in ND and KD groups. (**D**) qPCR analyses of IL-1, IL-1b, IL-8 and TNF-α gene expression. (**E**) Representative F4/80 IHC staining of tumors from each treatment group. Scale bars: 0.1 mm. The Mann–Whitney U test was used to compare two independent groups. *** $p < 0.001$ vs. black bar.

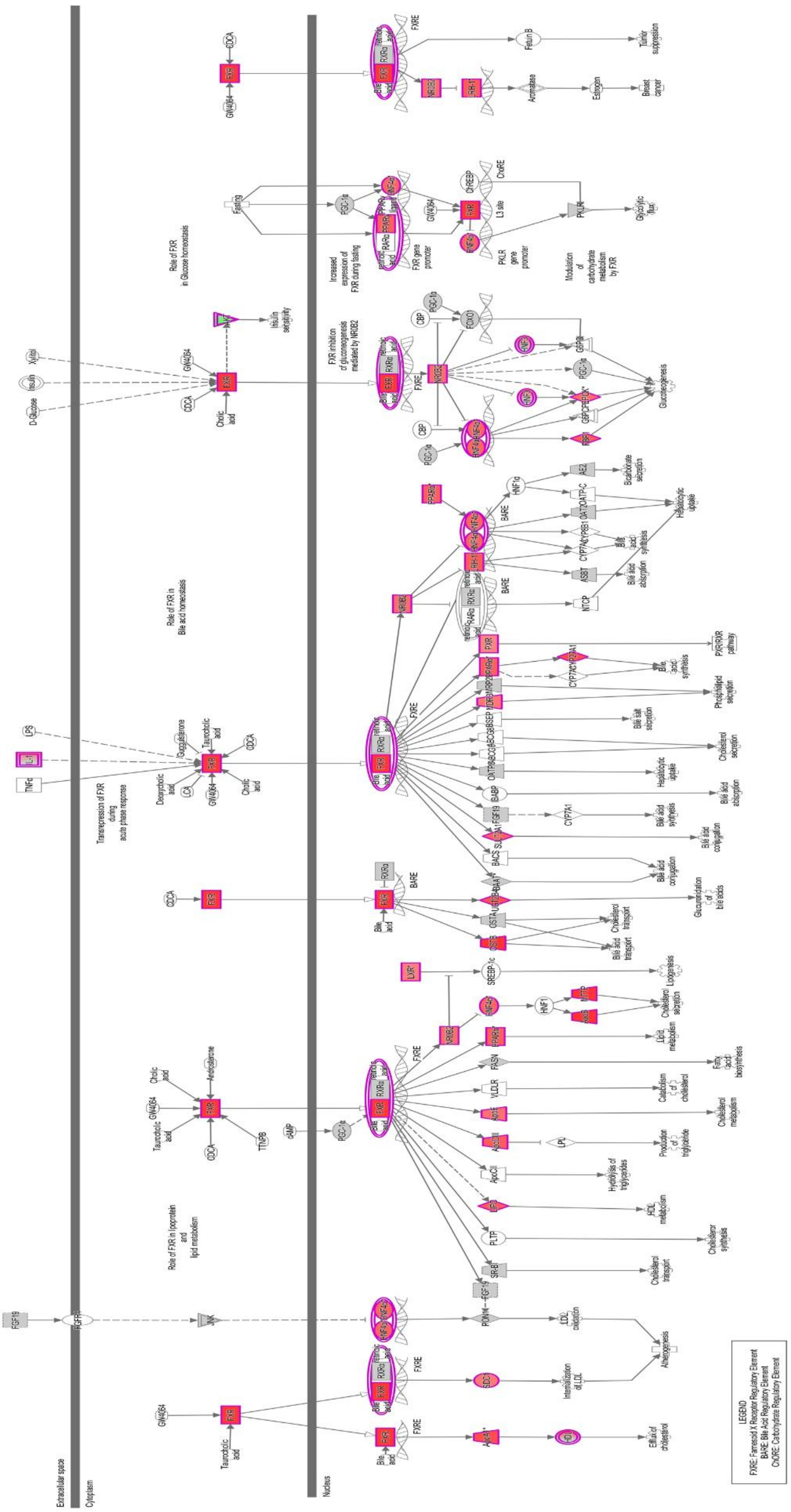

Figure S2. KEGG enrichment pathway: FXR/RXR activation**.** The up-regulation (red color shaded genes) of most genes in this pathway in Huh-7 shlacZ vs. Huh-7 shHMGCS2 cells.

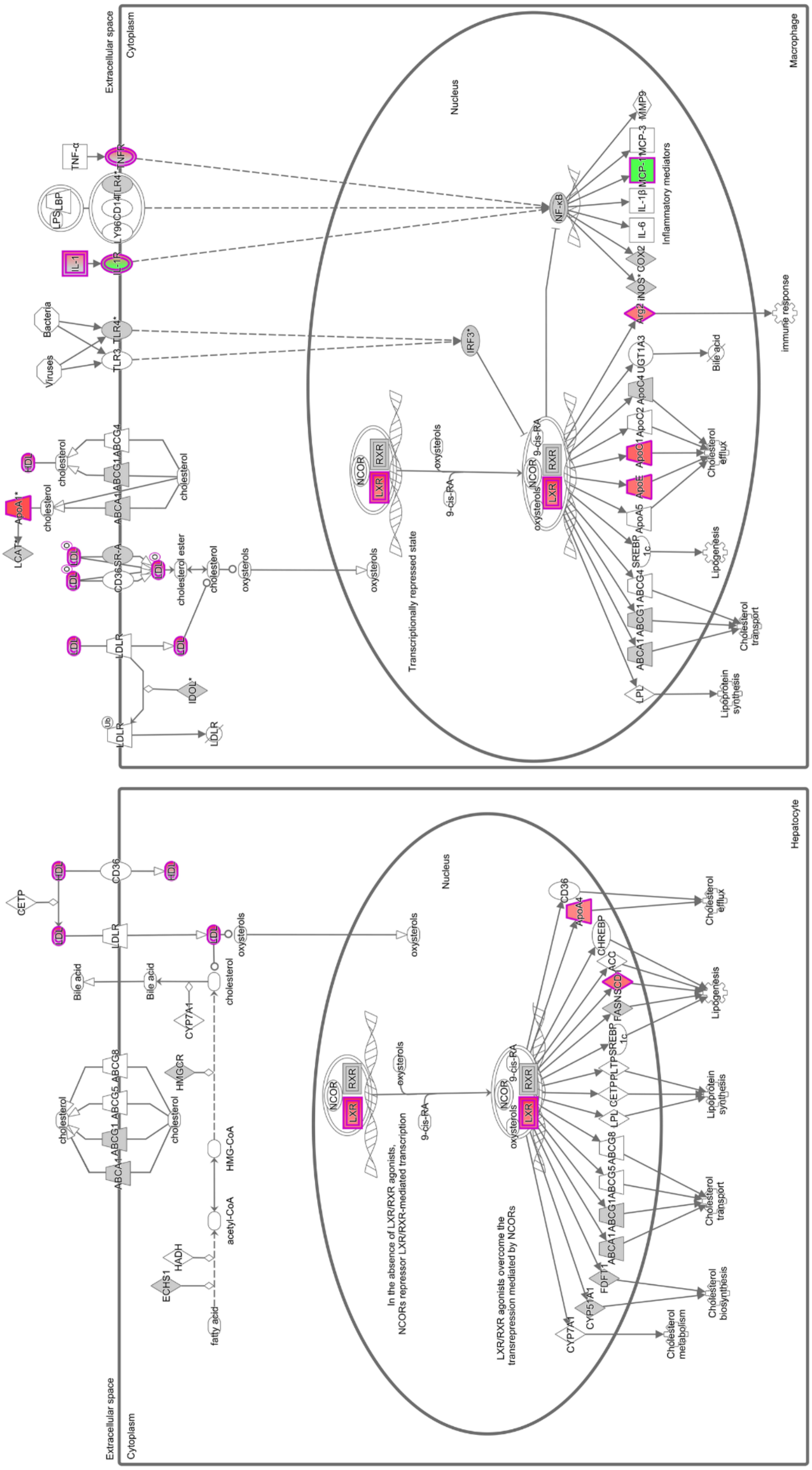

Figure S3. KEGG enrichment pathway: LXR/RXR activation. The downregulation (green color shaded genes) and up-regulation (red color shaded genes) of most genes in this pathway in Huh-7 shlacZ vs. Huh-7 shHMGCS2 cells.

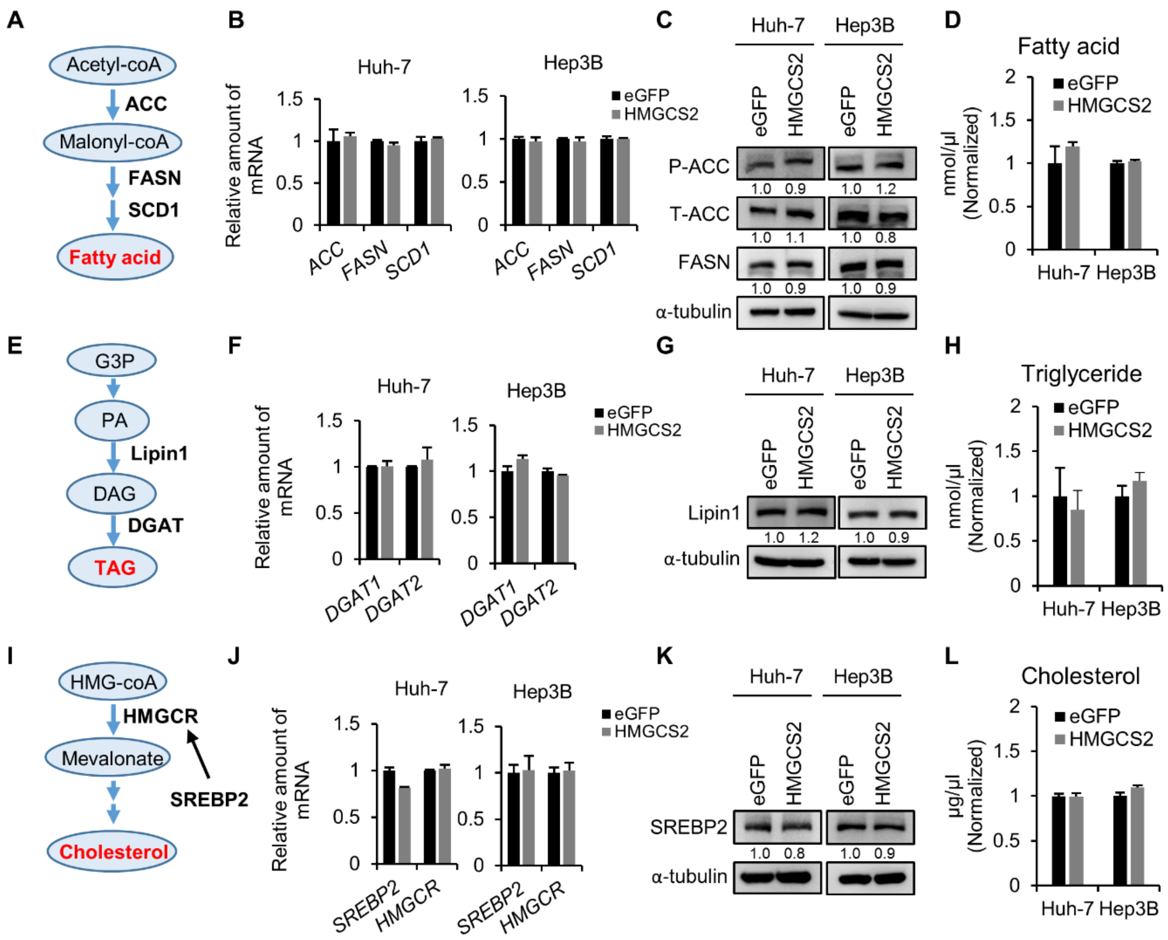

Figure S4. HMGCS2 overexpression did not affect fatty acid, triglyceride, and cholesterol synthesis in both Huh-7 and Hep3B cells. (**A**) Diagram of fatty acid synthesis. (**B**) qPCR analyses of ACC, FASN, and SCD1 gene expression. (**C**) Representative western blotting images of T/p-ACC, and FASN expression. (**D**) Intracellular fatty acids were measured using a colorimetric fatty acid quantification kit. (**E**) Diagram of triglyceride synthesis. (**F**) qPCR analyses of DGAT1 and DGAT2 gene expression. (**G**) Representative western blotting images of Lipin1 expression. (**H**) Intracellular triglyceride was confirmed by using a colorimetric triglyceride quantification kit. (**I**) Diagram of cholesterol synthesis. (**J**) qPCR analyses of SREBP2 and HMGCR gene expression. (**K**) Representative western blotting images of SREBP2 expression. (**L**) Intracellular cholesterol was measured by using a colorimetric cholesterol quantification kit. The quantification of the western blot images was presented by using ImageJ system and the whole blot images can be found in Figure S13.

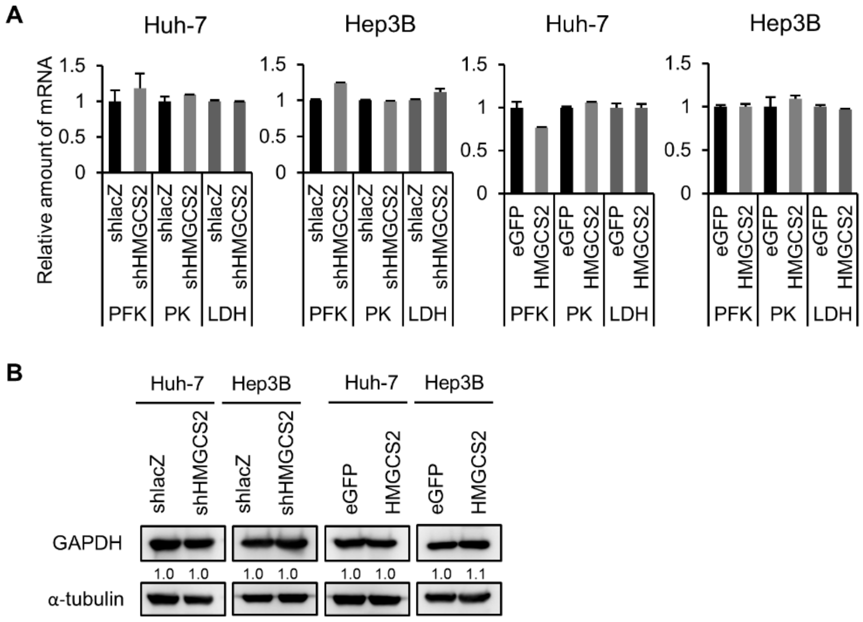

Figure S5. HMGCS2 expression did not affect the glycolysis signaling pathway in HCC cells**.** (**A**) Results from qPCR analyses of phosphofructokinase (PFK), pyruvate kinase (PK), and lactate dehydrogenase (LDH) gene expression in HMGCS2 knockdown and overexpressed Huh-7 and Hep3B cells. (B) Representative western blotting images of GAPDH expression. The quantification of the western blot images was presented by using ImageJ system and the whole blot images can be found in Figure S14.

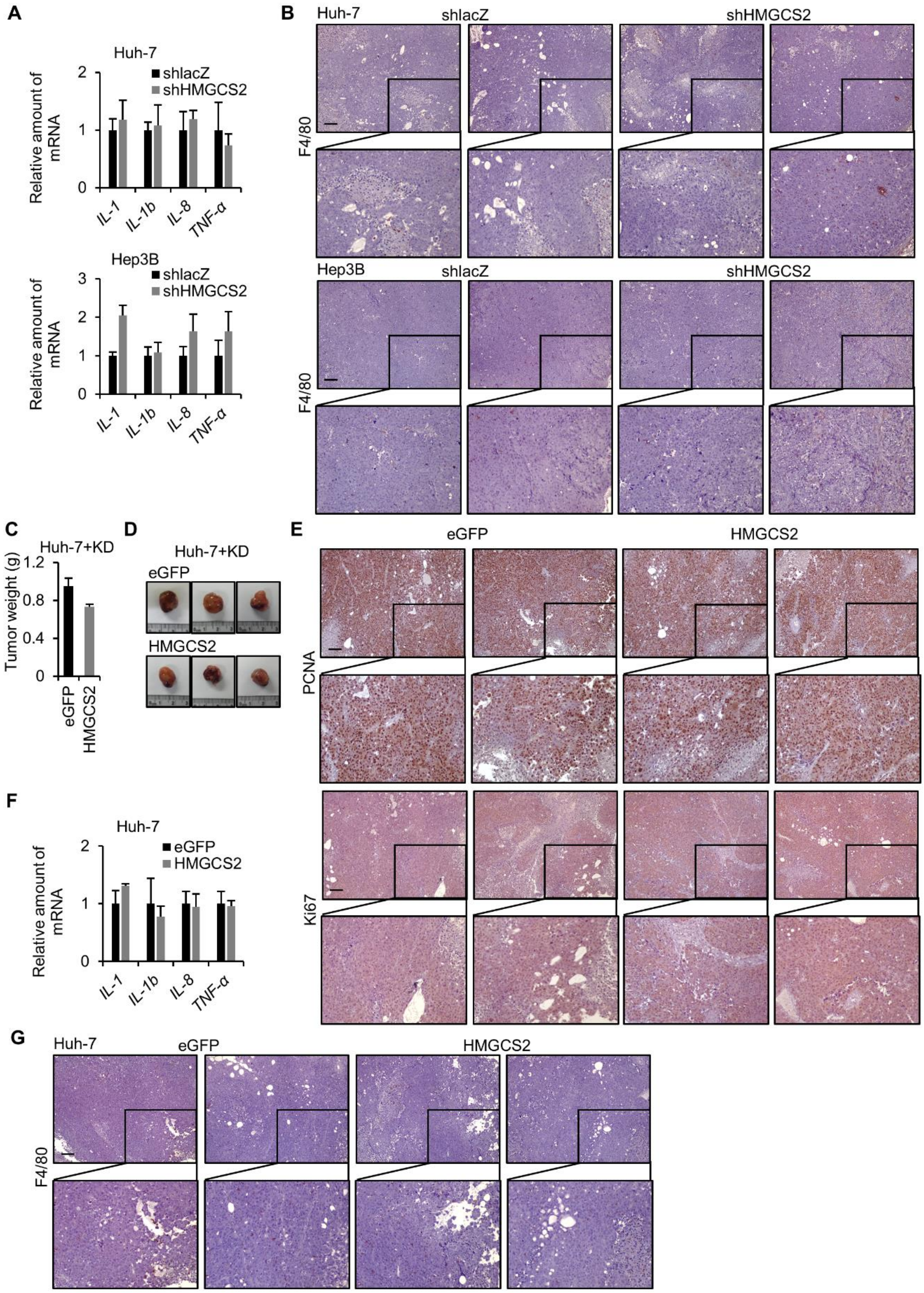

Figure S6. HMGCS2 overexpression did not affect tumor growth in KD-fed mice and HMGCS2 expression did not influence the tumor inflammation. (**A**) qPCR analyses of IL-1, IL-1b, IL-8 and TNF-α gene expression. (**B**) Representative F4/80 IHC staining of tumors from each treatment group. (**C**) The tumor weight from mice fed either an ND or KD. n = 5/group. (**D**) Representative images of tumors from each group. (**E**) Representative PCNA and Ki67 IHC staining of tumors from each treatment group. (**F**) qPCR analyses of IL-1, IL-1b, IL-8 and TNF-α gene expression. (**G**) Representative F4/80 IHC staining of tumors from each treatment group. Scale bars: 0.1 mm.

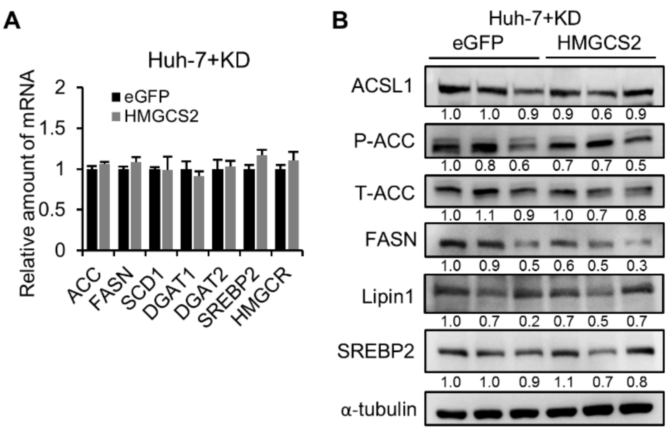

Figure S7. HMGCS2-overexpressed tumors did not influence expression of lipogenesis-related markers under KD feeding. (**A**) qPCR analyses of ACC, FASN, SCD1, DGAT1, DGAT2, SREBP2, and HMGCR gene expression and (**B**) western blot analyses of ACSL1, T/P-ACC, FASN, Lipin1, and SREBP2 expression of KD fed eGFP and HMGCS2 tumors. The quantification of the western blot images was presented by using ImageJ system and the whole blot images can be found in Figure S15.

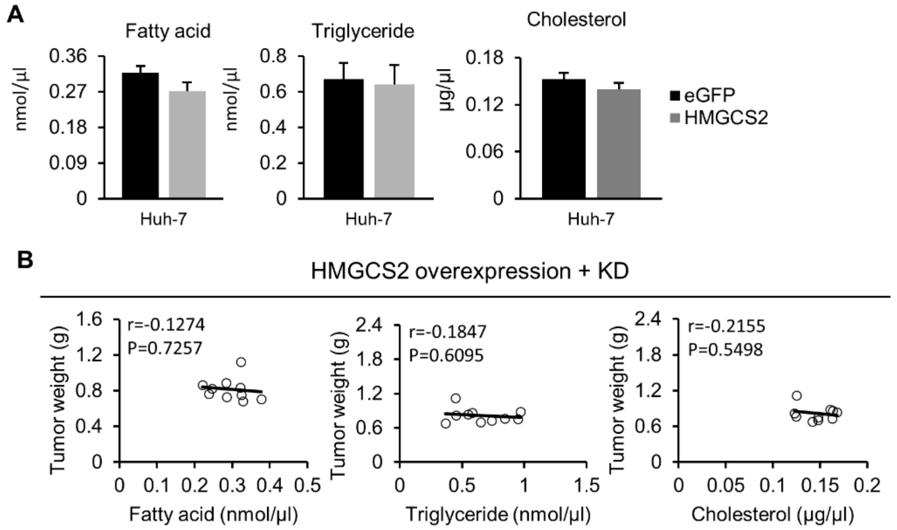

Figure S8. HMGCS2-overexpressed tumors did not alter lipid content and showed no correlation with tumor weight. (**A**) The fatty acids, triglycerides, and cholesterol in the tumor mass. (**B**) Pearson correlation between each lipid content and tumor weight in the indicated groups.

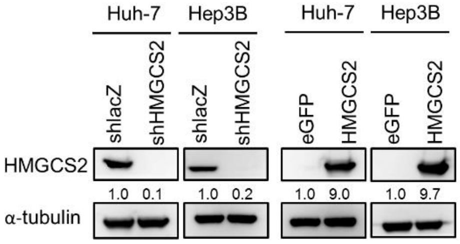

Figure S9. Establishment of cell lines with stable HMGCS2 overexpression and knockdown. Representative western blotting images of HMGCS2 expression. The quantification of the western blot images was presented by using ImageJ system and the whole blot images can be found in Figure S16.

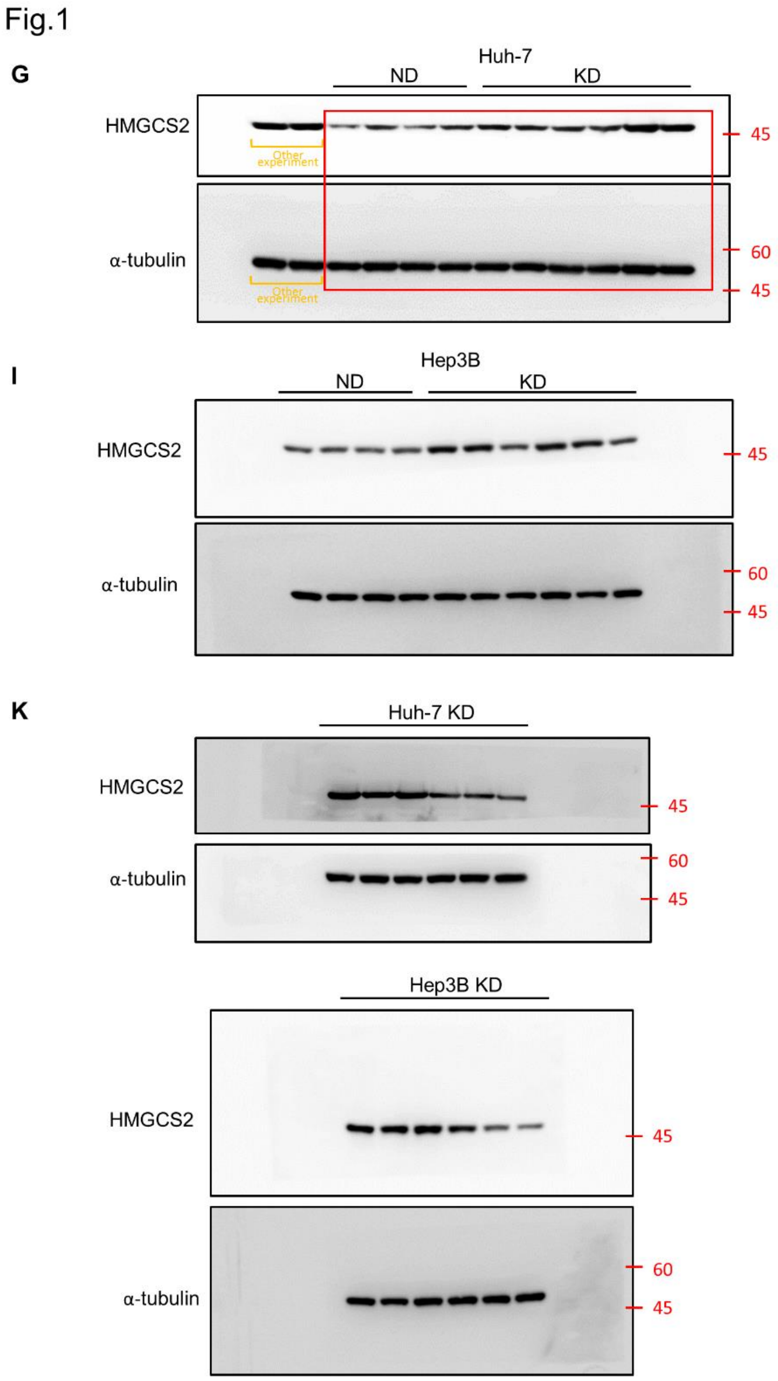

Figure S10. Original unedited pictures and protein quantification of Figure 1**.** Complete western blot image corresponding to Figure 1G,I,K showing all bands and molecular weight markers for HMGCS2 and α-tubulin.

Fig.3

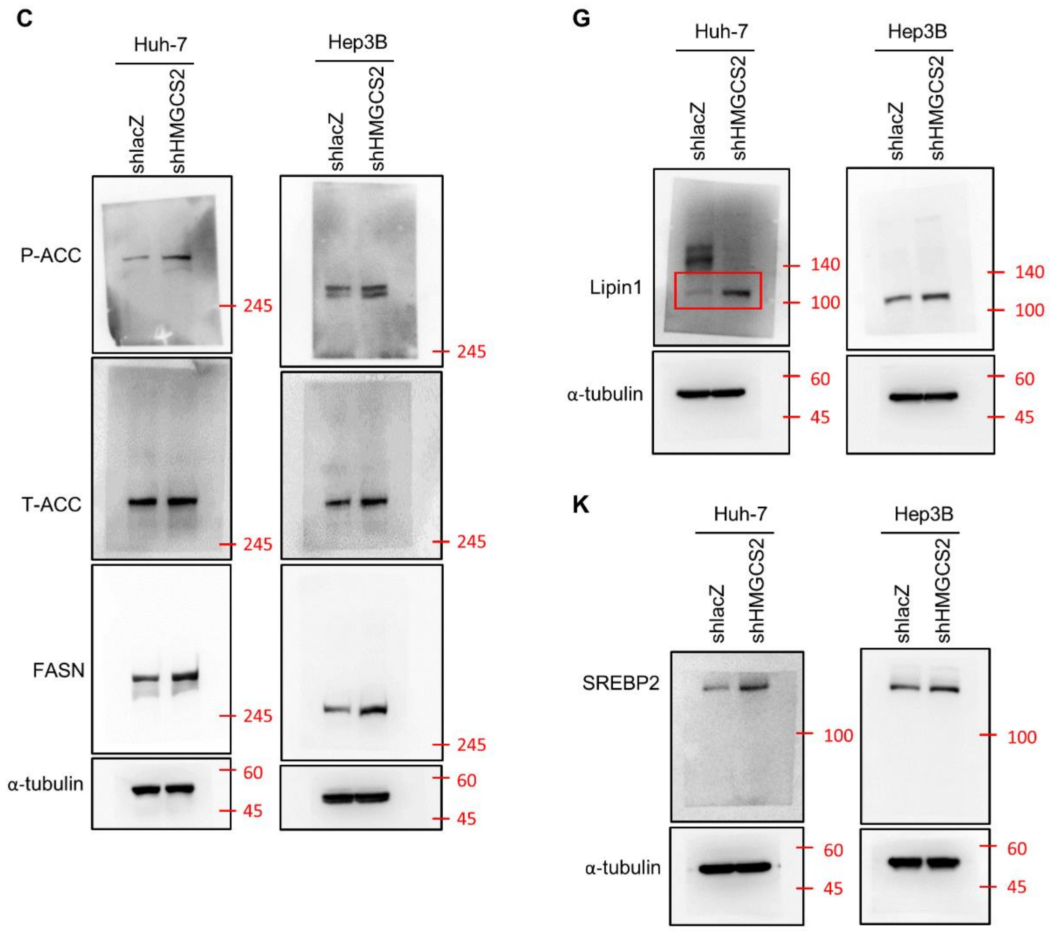

Figure S11. Original unedited pictures and protein quantification of Figure 3. Complete western blot image corresponding to Figure 3C,G,K showing all bands and molecular weight markers for T/P-ACC, FASN, Lipin1, SREBP2, and α-tubulin.

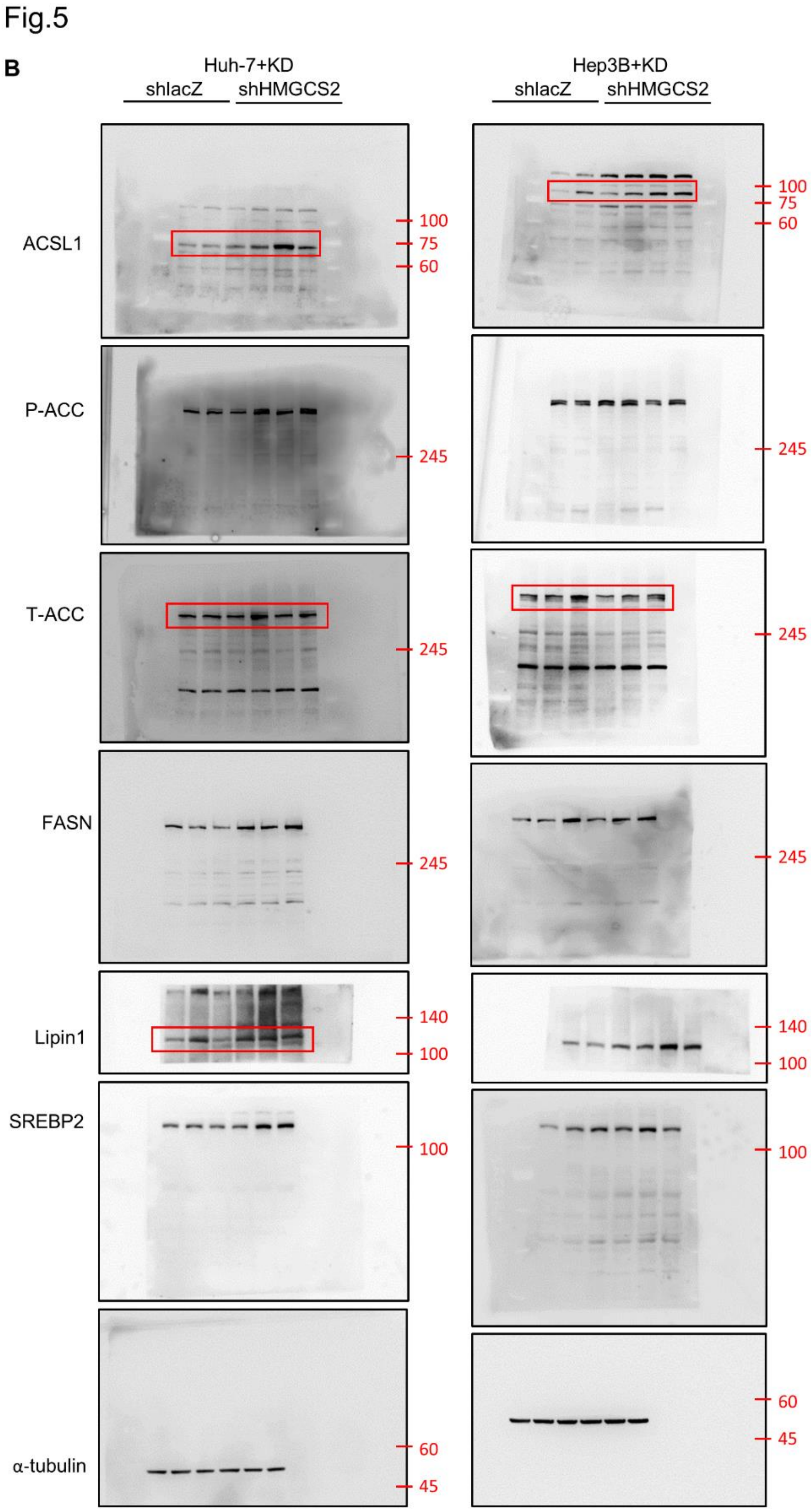

Figure S12. Original unedited pictures and protein quantification of Figure 5. Complete western blot image corresponding to Figure 5B showing all bands and molecular weight markers for ACSL1, T/P-ACC, FASN, Lipin1, SREBP2, and α-tubulin.

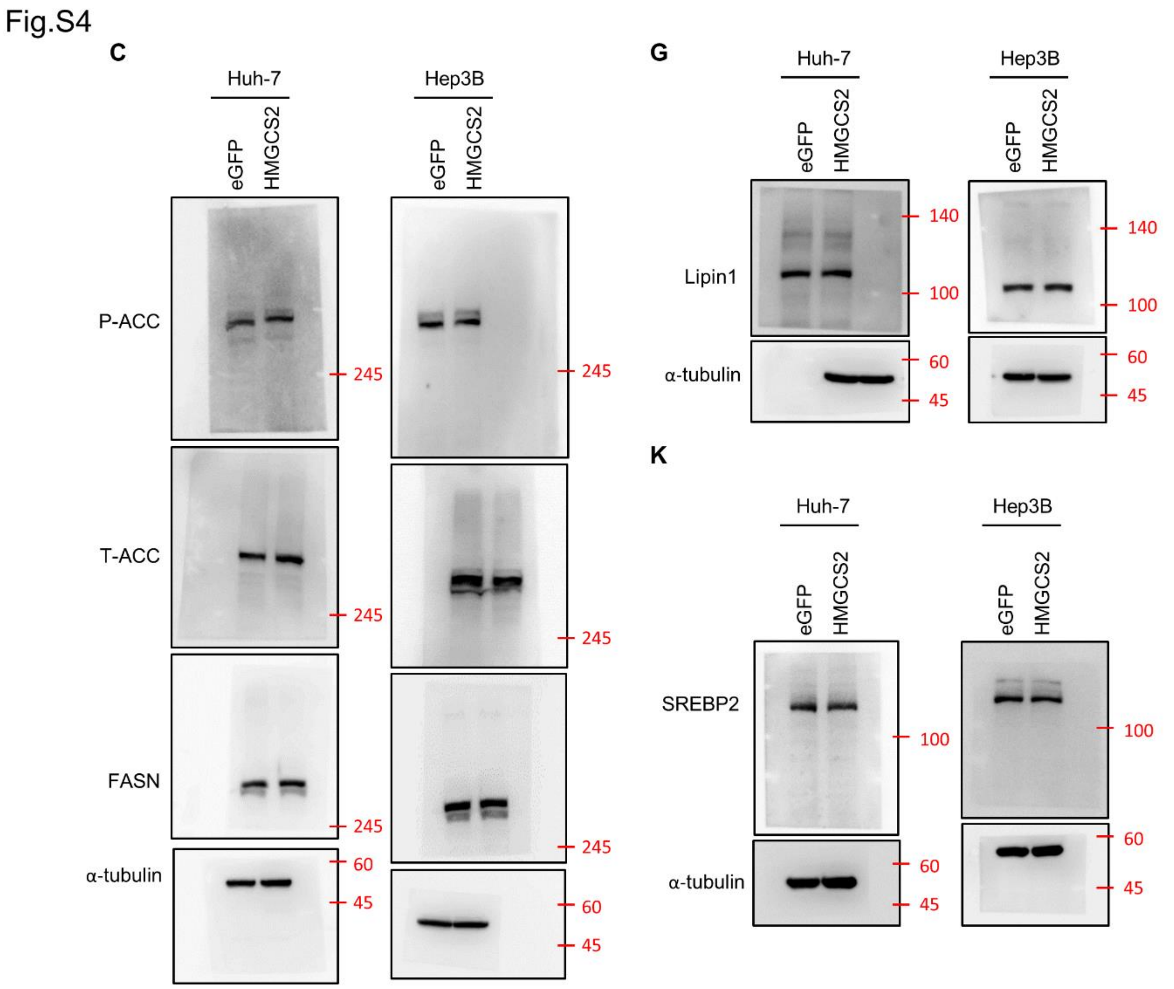

Figure 13. Original unedited pictures and protein quantification of Figure S4. Complete western blot image corresponding to Figure S4C,G,K showing all bands and molecular weight markers for T/P-ACC, FASN, Lipin1, SREBP2, and α-tubulin.

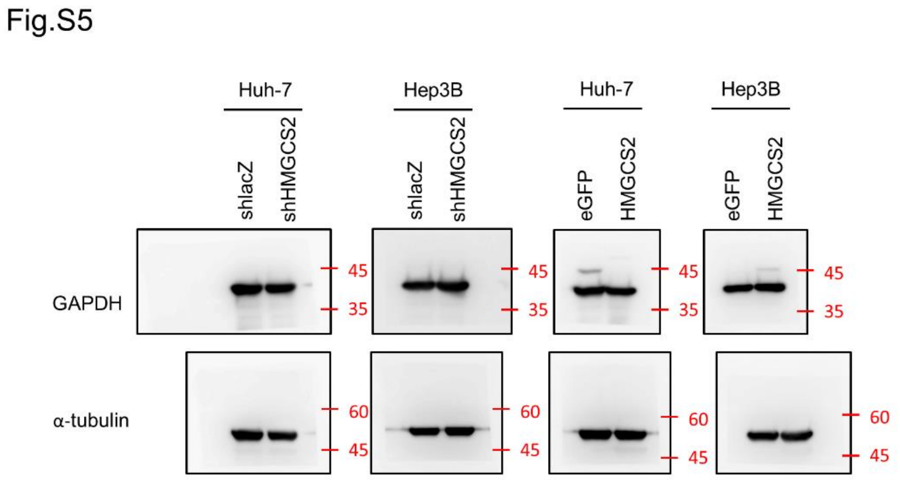

Figure S14. Original unedited pictures and protein quantification of Figure S5. Complete western blot image corresponding to Figure S5B showing all bands and molecular weight markers for GAPDH and α-tubulin.

Fig.S7

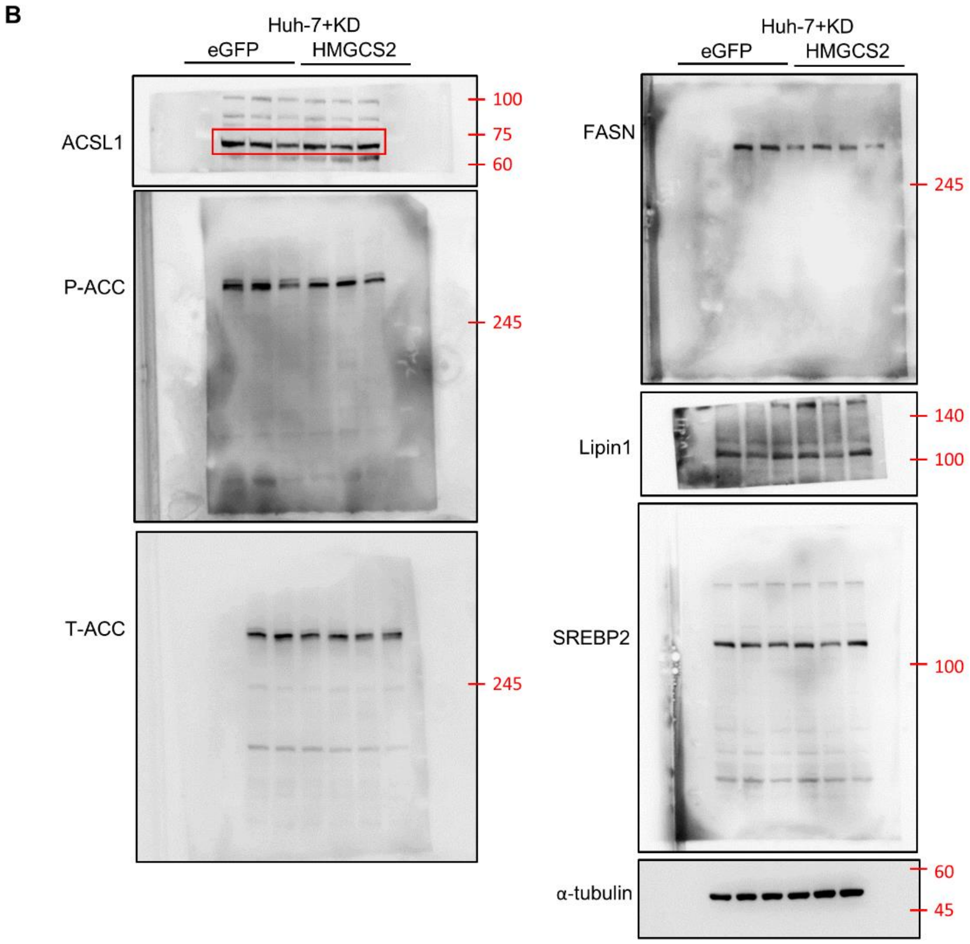

Figure S15. Original unedited pictures and protein quantification of Figure S7. Complete western blot image corresponding to Figure S7B showing all bands and molecular weight markers for ACSL1, T/P-ACC, FASN, Lipin1, SREBP2, and α-tubulin.

Fig.S9

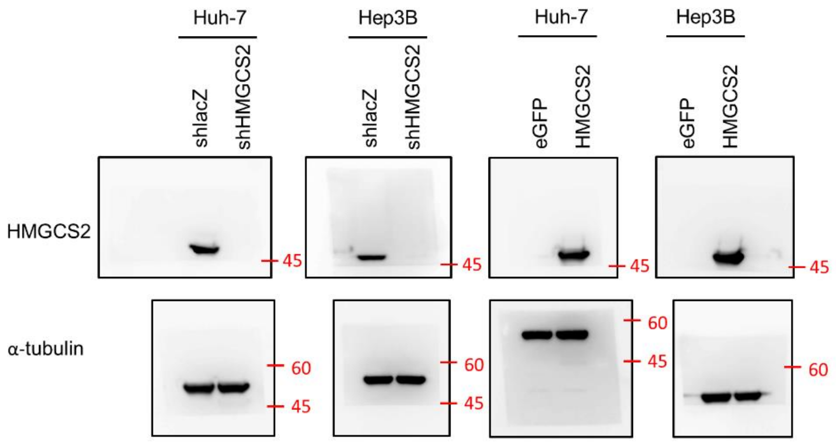

Figure S16. Original unedited pictures and protein quantification of Figure S9. Complete western blot image corresponding to Figure S9 showing all bands and molecular weight markers for HMGCS2 and α-tubulin.
